# Using Stochastic Causal Trees to Augment Bayesian Networks for Modeling eQTL Datasets

**DOI:** 10.1186/1471-2105-12-7

**Published:** 2011-01-06

**Authors:** Kyle C Chipman, Ambuj K Singh

**Affiliations:** 1 Biomolecular Science and Engineering Program, UC Santa Barbara, Santa Barbara, CA, USA; 2 Department of Computer Science, UC Santa Barbara, Santa Barbara, CA, USA

## Abstract

**Background:**

The combination of genotypic and genome-wide expression data arising from segregating populations offers an unprecedented opportunity to model and dissect complex phenotypes. The immense potential offered by these data derives from the fact that genotypic variation is the sole source of perturbation and can therefore be used to reconcile changes in gene expression programs with the parental genotypes. To date, several methodologies have been developed for modeling eQTL data. These methods generally leverage genotypic data to resolve causal relationships among gene pairs implicated as associates in the expression data. In particular, leading studies have augmented Bayesian networks with genotypic data, providing a powerful framework for learning and modeling causal relationships. While these initial efforts have provided promising results, one major drawback associated with these methods is that they are generally limited to resolving causal orderings for transcripts most proximal to the genomic loci. In this manuscript, we present a probabilistic method capable of learning the causal relationships between transcripts at all levels in the network. We use the information provided by our method as a prior for Bayesian network structure learning, resulting in enhanced performance for gene network reconstruction.

**Results:**

Using established protocols to synthesize eQTL networks and corresponding data, we show that our method achieves improved performance over existing leading methods. For the goal of gene network reconstruction, our method achieves improvements in recall ranging from 20% to 90% across a broad range of precision levels and for datasets of varying sample sizes. Additionally, we show that the learned networks can be utilized for expression quantitative trait loci mapping, resulting in upwards of 10-fold increases in recall over traditional univariate mapping.

**Conclusions:**

Using the information from our method as a prior for Bayesian network structure learning yields large improvements in accuracy for the tasks of gene network reconstruction and expression quantitative trait loci mapping. In particular, our method is effective for establishing causal relationships between transcripts located both proximally and distally from genomic loci.

## Background

In order to model and dissect the complexity underlying physiological processes, including diseases, developmental programs, and responses to pharmacological treatments, systematic approaches based on genome-wide data are imperative. Expression profiling technologies, such as microarray [1,2] and RNA-Seq platforms [3], provide quantification of mRNA levels on a genome-wide scale, prompting computational methods aimed at learning a more holistic perspective of cellular processes. Parallel advancements in the area of genotypic profiling, including high-throughput sequencing and SNP detection, offer information complementary to that of expression data. These concurrent developments pave the way for genetical genomic studies, which provide the joint space of expression and genotypic data corresponding to offspring that arise from a segregating population [4]. To date, eQTL datasets have been published for several organisms [5-10], providing ample opportunity to develop novel computational methodologies. The tandem existence of expression and genotypic data is especially powerful in that it allows one to reconcile changes in expression programs in the context of the specific genetic combinations represented by the offspring. Since natural genetic variation is the sole source of perturbation, it is logical to view genomic loci as epicenters of phenotypic variation in eQTL-derived causal networks. Consequently, modeling eQTL datasets enables one to hypothesize on how genotypic variation results in phenotypic changes.

Already several studies have provided methodologies aimed at exploiting the genotypic component of eQTL data to improve causal modeling in gene networks [9,11-16]. Bing *et al. *introduced methodology to build directed networks starting from a set of candidate cis-genes for each locus [14], establishing directed edges from candidate cis-genes to distally-located genes. This approach yields local regulatory models for individual loci, and the authors also present an innovative approach based on partial correlations to identify models where two regulators play complementary roles in controlling a common set of genes. The methodology of Bing *et al. *was later applied to an eQTL dataset representing *Arabidopsis *by Keurentjes *et al.*, who also incorporated information regarding DNA sequence to improve the estimation of cis-genes [9]. While this application was successful in providing hypotheses regarding local regulatory models, it does not resolve causal orderings amongst distally located transcripts. Furthermore, modeling local regulatory programs with respect to individual loci leaves room for improvement in the sense that each of the respective models are disjoint. A worthwhile goal is to produce more holistic and systematic methodologies capable of modeling the complex interdependencies between multiple loci and transcripts. Indeed, it has been estimated that the genetic basis of many transcripts is extensively complex, with upwards of 50% of transcripts being linked to five or more loci [17]. The need for a comprehensive and systematic approach was addressed by Schadt and colleagues, who developed a novel method to augment Bayesian networks with probabilistic measures to direct causal orderings of gene pairs with respect to genomic loci [11-13,18]. Their method, which is based on a conditional bivariate normal model, determines if two transcripts linked to a common locus are best modeled as causal or independent [12]. Ultimately, the information generated by their method is incorporated as a prior for Bayesian network structure learning [19]. This approach has yielded promising results when applied to yeast [13] and mouse [12], providing hypotheses regarding the architecture of eQTL networks. Furthermore, the authors published a study on synthetic networks to quantify the performance gains associated with their method [18] as compared to standard Bayesian network structure learning. While their method proved efficacious at resolving causal orientations between correlated transcripts in the context of a global network, the scope is generally limited to the upper echelons of the causal hierarchy, an attribute that stems from their reliance on using genomic loci as causal anchors. Ideally, one could commence at the genomic loci, learn the causal orderings of the most proximal transcripts, then advance down the causal hierarchy propagating the structural information gleaned from the upper levels of the network. The probabilistic method presented herein realizes this concept by stochastically reconstructing the causal hierarchy, which is subsequently incorporated as a prior into Bayesian network structure learning.

A distinct but related problem to gene network reconstruction (GNR) is expression quantitative trait loci (eQTL) mapping. While these two tasks have to date been addressed independently, they are likely to become more intertwined as eQTL-related computational methodologies advance. This corollary follows from the fact that accurately-modeled networks should inform on transcript-locus associations by virtue of the implied causal pathways. Traditional univariate methods, which involve an exhaustive search between all transcripts and loci, typically entail the use of linear regression, ANOVA, or the t-test [20], where the chosen statistic assesses the extent to which a trait (transcript level) is linked to a locus. While straightforward, this approach results in a considerable loss in statistical power due to the need to correct for multiple hypothesis testing. The issue of multiple testing is compounded when transcripts are tested for simultaneous linkage to two loci, where an exhaustive search across all loci results in complexity that is quadratic in the number of loci. Addressing this limitation, Storey *et al. *developed a stepwise mapping procedure [21] whereby testing linkage to a secondary locus involves conditioning on the primary locus, reducing the complexity to 2*L *- 1 tests, where L is the number of loci. This approach, which controls for confounding linkages and tests for epistasis, was later generalized for multiple interval mapping by Zou *et al. *[22]. While stepwise mapping techniques produce notable improvements over exhaustive searches, they do not take advantage of the fact that many transcripts sharing linkages to common loci exhibit strong correlation structure. Suitably, recent methods have focused on mapping multiple (correlated) traits simultaneously to genomic loci [23-26]. Pan *et al. *[25], Litvin *et al. *[24] and Lee *et al. *[23] developed methods based on penalized regression frameworks to discover modules that can be explained by common expression programs. Similarly, Zhang *et al. *developed a Bayesian partition method to learn modules of transcripts sharing linkage to common genomic loci [26]. These clustering based methods offer the distinct advantage of using fewer parameters while detecting eQTL linkages, however, they do not attempt to reconstruct the causal hierarchy and are therefore limited in the biological hypotheses that they offer.

Our goal is to reconstruct causal networks with high-fidelity at all levels of the network. Consequently, by improving the accuracy of the reconstructed network, we show that our method can provide biological hypotheses as well as enable greater accuracy in eQTL mapping.

## Results

We assess the performance of our method for the tasks of gene network reconstruction (GNR) and expression quantitative trait-loci (eQTL) mapping. For GNR, we compare our methodology to standard unaugmented Bayesian network structure learning, herein referred to as "Basic," and the leading LCMS methodology of Schadt and colleagues [11-13], herein referred to as "LCMS." We refer to our stochastic causal tree method as "SCT." For eQTL mapping, we compare our method to traditional univariate mapping. Several statistical methods exist that are suitable for implementing univariate mapping, including regression, ANOVA and the t-test [20]. Since our study involves data that are generated from Gaussian functions, we utilize the t-test in lieu of non-parametric alternatives such as the Wilcoxon rank-sum test. We generated a synthetic network according to the protocol outlined in a previous eQTL study [18] (see Methods section). The synthetic network is composed of 2, 200 transcripts and 50 loci, connected by 2, 598 edges. A total of six datasets are generated from the network adjacency matrix by regression models with noise (see Methods section). The first three eQTL datasets consist of 100, 200, and 300 samples, respectively, and are characterized by relatively strong correlation structure. We herein refer to these as "strongly correlated networks" or "datasets composed of stronger correlation structure." The three strongly correlated networks feature a mean correlation between parent and child of 0.68. However, it is possible that modeling techniques are biased in discovering stronger links, and weaker interactions may be more prevalent than can be detected, a point that has been made in previous studies [18]. In consideration of this possibility, we simulated three parallel datasets consisting of 100, 200, and 300 samples with weaker correlation structure. The three datasets with weaker correlation structure possess a mean correlation between parent and child of 0.55. Table 1 in the Methods section provides a summary of the statistics characterizing the datasets and the parameters used to generate the data. Our goal was to assess performance on datasets representing what we feel is a reasonable estimate of lower and upper ranges of interaction strengths in gene networks.

**Table 1:** Network Parameters

	Stronger Cor. Structure	Weaker Cor. Structure
Mean CC	0.68	0.55
90% Upper Interval	0.88	0.76
90% Lower Interval	0.45	0.31

Mean *a_i_*	0.75	0.6
S.D. *a_i_*	0.2	0.2
Mean *k_i_*	0.5	0.5
S.D. *k_i_*	0.1	0.1

### Precision and recall of network edges

**Figure 1:**
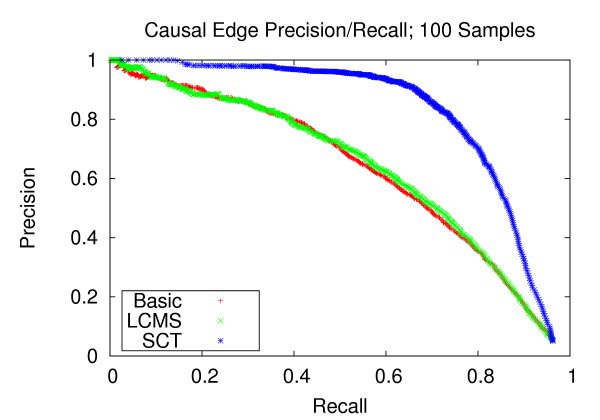
**Causal Edge Precision-Recall; 100 Samples**. Precision and recall of directed edges for 100 experiments. At the precision level of 0.8, the SCT method yields a recall of 0.744, versus 0.391 and 0.393 for the LCMS and Basic methods, respectively.

**Figure 2:**
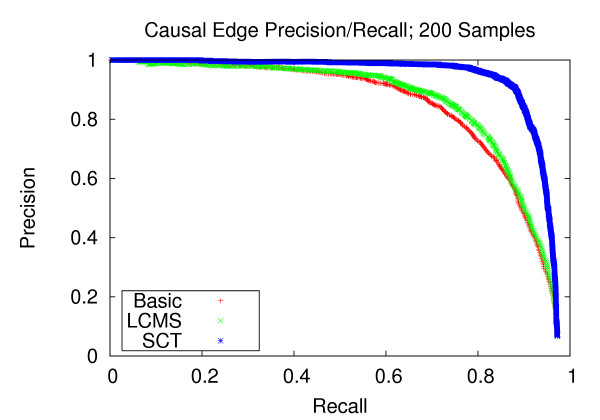
**Causal Edge Precision-Recall; 200 Samples**. Precision and recall of directed edges for 200 experiments. At the precision level of 0.8, the SCT method produces a recall of 0.910, versus 0.789 and 0.753 for the LCMS and Basic methods, respectively.

**Figure 3:**
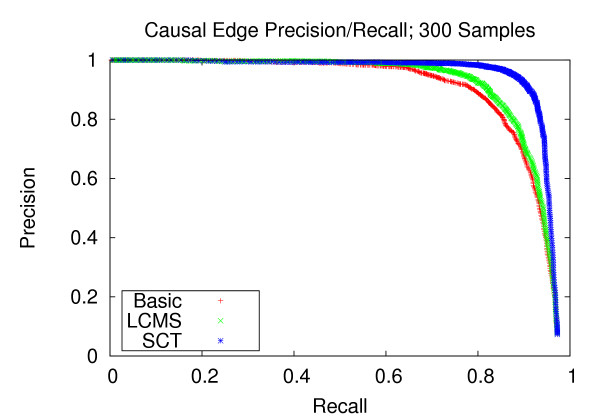
**Causal Edge Precision-Recall; 300 Samples**. Precision and recall of directed edges for 300 experiments. At the precision level of 0.8, the SCT method produces a recall of 0.940, versus 0.876 and 0.856 for the LCMS and Basic methods, respectively.

We first assess the performance of gene network reconstruction. Figures 1, 2 and 3 refer to the networks composed of stronger correlation structure, and show that our method provides appreciable performance gains in terms of precision and recall. For the dataset with 100 experiments, our method achieves a recall of 0.744 at a precision of 0.8. Comparatively, the LCMS method achieves a recall of 0.391 at the same level of precision. For the dataset with 200 experiments, our method achieves a recall of 0.944 at a precision of 0.8, whereas the LCMS method yields a recall of 0.872 at the same level of precision. Finally, for 300 experiments, the precision level of 0.8 corresponds to recall levels of 0.949 and 0.875 for the SCT and LCMS methods, respectively. Our method, when applied to 200 experiments, outperforms unaugmented Bayesian network structure learning when applied to 300 experiments.

**Figure 4:**
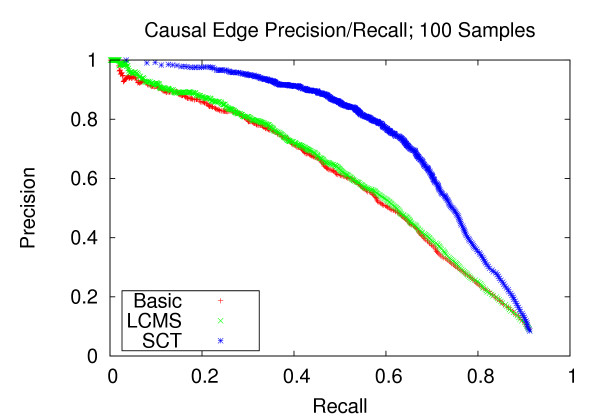
**Causal Edge Precision-Recall w/Weaker Correlations; 100 Samples**. Precision and recall of directed edges for 100 experiments from datasets with weaker correlation structure. At the precision level of 0.8, the SCT method yields a recall of 0.570, versus 0.310 and 0.300 for the LCMS and Basic methods, respectively.

**Figure 5:**
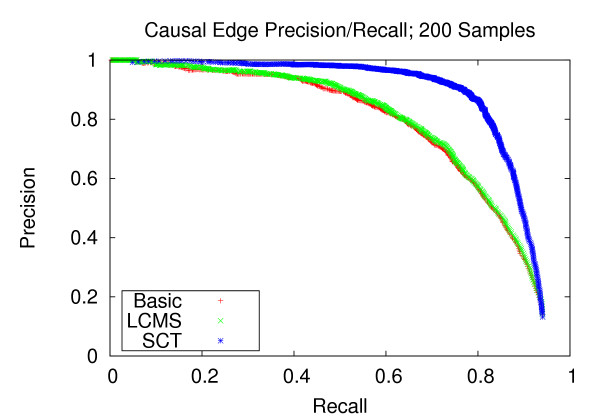
**Causal Edge Precision-Recall w/Weaker Correlations; 200 Samples**. Precision and recall of directed edges for 200 experiments from datasets with weaker correlation structure. At the precision level of 0.8, the SCT method produces a recall of 0.824, versus 0.640 and 0.637 for the LCMS and Basic methods, respectively.

**Figure 6:**
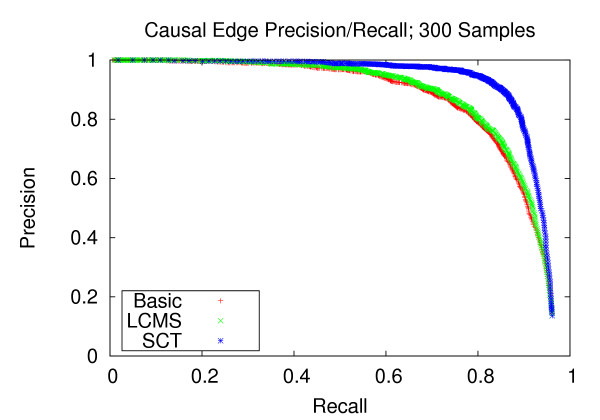
**Causal Edge Precision-Recall w/Weaker Correlations; 300 Samples**. Precision and recall of directed edges for 300 experiments from datasets with weaker correlation structure. At the precision level of 0.8, the SCT method produces a recall of 0.894, versus 0.810 and 0.793 for the LCMS and Basic methods, respectively.

Figures 4, 5 and 6 provide a performance comparison for eQTL datasets with weaker correlation structure. The results are consistent with those corresponding to the strongly correlated datasets. SCT-augmented structure learning yields improvements in recall over unaugmented structure learning for a broad range of precision levels, particularly between 0.6 and 0.8.

We note that the performance gains of LCMS-augmented structure learning over unaugmented structure learning reported by Zhu *et al. *[18] are recapitulated in our experiments, though the margin of improvement is slightly lower in our study. This is due to the fact that we explicitly model genomic loci as head nodes, which equates to the authors' logic whereby transcripts with cis-linkages are required to be head nodes. Consequently, the performances of the baseline "Basic" Bayesian networks are higher in our study as compared to Zhu *et al. *[18]. In experiments not published herein, we estimate that the performance margins between the Basic and LCMS-augmented methods are generally at least twice as large when this logic is not included into the unaugmented Bayesian network structure learning procedure, indicating that performance gains are realized by simply establishing the head nodes as such.

### Precision and recall of transcript-locus associations

Next, we sought to assess the potential of using the learned networks for the purpose of expression quantitative trait loci (eQTL) mapping. In order to establish a transcript-locus linkage, for each head node (locus) in the Bayesian network, we run a depth-first search down the respective branches. All reachable transcripts from the source locus are associated with that locus. Starting with a set of sampled networks, we apply this procedure on each of the individual networks, each yielding a set of transcript-loci linkages. Precision-recall curves were generated from the totality of the individual networks (Methods section). We compare this approach to traditional univariate mapping utilizing a t-test. We note that while there are several recent clustering-based methodologies related to eQTL mapping [23-26], they do not attempt to reconstruct gene networks. Since GNR is our primary goal, we restrict our analysis to the three versions of Bayesian networks and traditional univariate mapping.

**Figure 7:**
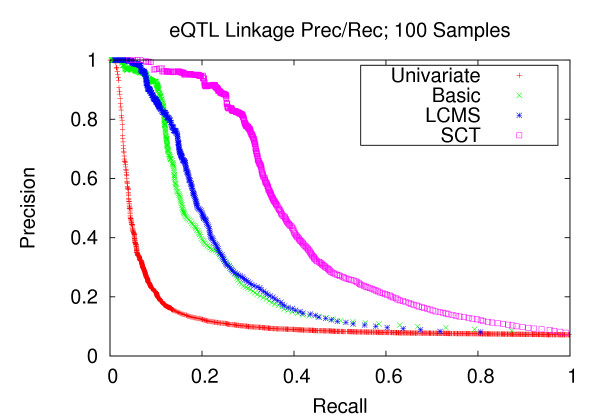
**eQTL Linkage Precision-Recall; 100 Samples**. Precision and recall of transcript-locus linkages for 100 experiments. At the precision level of 0.8, the SCT method achieves a recall of 0.290, versus 0.130, 0.118 and 0.03 for the LCMS, Basic and univariate mapping methods, respectively.

**Figure 8:**
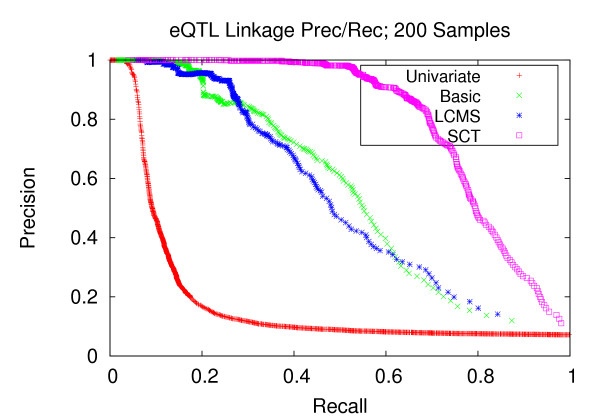
**eQTL Linkage Precision-Recall; 200 Samples**. Precision and recall of transcript-locus linkages for 200 experiments. At the precision level of 0.8, the SCT method achieves a recall of 0.695, versus 0.301, 0.345 and 0.065 for the LCMS, Basic and univariate mapping methods, respectively.

**Figure 9:**
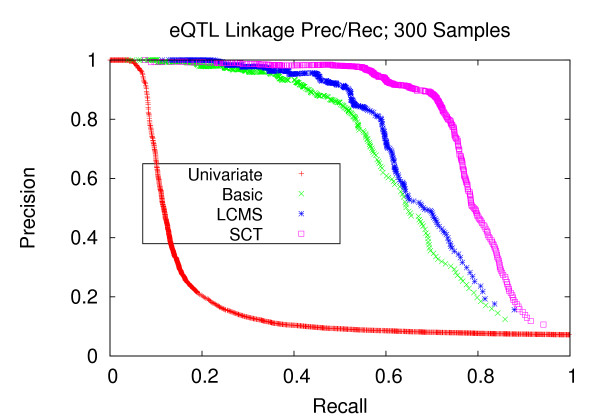
**eQTL Linkage Precision-Recall; 300 Samples**. Precision and recall of transcript-locus linkages for 300 experiments. At the precision level of 0.8, the SCT method achieves a recall of 0.735, versus 0.590, 0.25 and 0.08 for the LCMS, Basic and univariate mapping methods, respectively.

**Figure 10:**
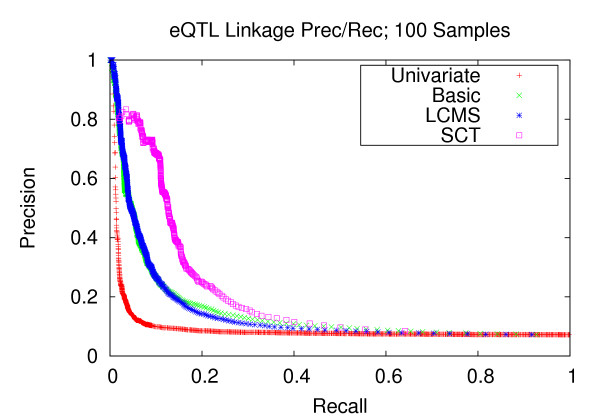
**eQTL Linkage Precision-Recall w/Weaker Correlations; 100 Samples**. Precision and recall of transcript-locus linkages for 100 experiments from datasets with weaker correlation structure. At the precision level of 0.8, the SCT method achieves a recall of 0.021 versus 0.020 and 0.019 for the LCMS and Basic methods, respectively. The univariate mapping method recall is 0.008.

**Figure 11:**
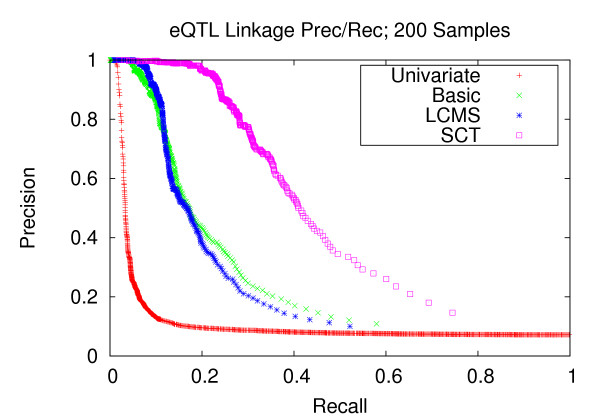
**eQTL Linkage Precision-Recall w/Weaker Correlations; 200 Samples**. Precision and recall of transcript-locus linkages for 200 experiments from datatsets with weaker correlation structure. At the precision level of 0.8, the SCT method achieves a recall of 0.282, versus 0.115, 0.110 and 0.025 for the LCMS, Basic and univariate mapping methods, respectively.

**Figure 12:**
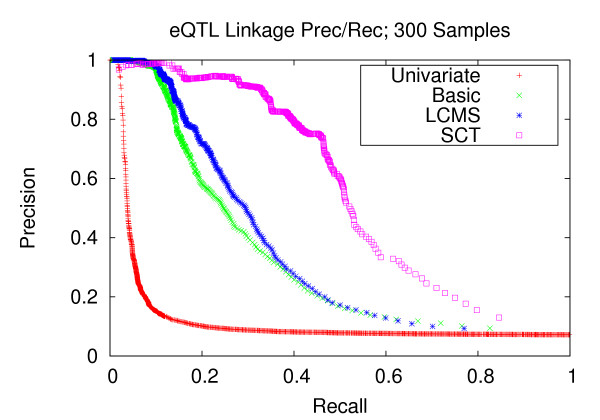
**eQTL Linkage Precision-Recall w/Weaker Correlations; 300 Samples**. Precision and recall of transcript-locus linkages for 300 experiments from datasets with weaker correlation structure. At the precision level of 0.8, the SCT method achieves a recall of 0.400, versus 0.164, 0.142 and 0.028 for the LCMS, Basic and univariate mapping methods, respectively.

Figures 7, 8, and 9 depict the performance enhancements associated with Bayesian networks as compared to traditional univariate mapping for the datasets with stronger correlation structure and containing 100, 200, and 300 samples. All three methods utilizing Bayesian networks resulted in performance gains over traditional univariate mapping. However, the performance gains associated with the SCT-augmented Bayesian networks were greater, providing 10-fold gains in recall in the precision range of 0.6 to 0.8. Performance gains for datasets with weaker correlation structure (Figures 10, 11, and 12) are largely consistent with those obtained on datasets composed of stronger correlation structure, though the performance on the dataset composed of 100 samples (Figure 10) only provides a roughly 3-fold gain over traditional univariate mapping.

### Robustness of network reconstruction

As outlined in the Methods section, we assess convergence and reconstruct consensus networks from two independent MCMC runs, each consisting of 150 million iterations. However, an additional test for robustness involves assessing the stability of edge frequencies across multiple MCMC runs. We follow the protocol outlined by Zhu *et al. *[18], whereby all edges above a predefined frequency threshold are extracted for five individual MCMC runs. Additional file 1 figures *S*1*a*-*S*1*c *provide the network reconstruction stabilities for datasets of weaker correlation structure and 200 samples, presented for each of the three methods: (a) unaugmented, (b) LCMS-augmented, and (c) SCT-augmented Bayesian networks. We adjusted the frequency threshold that determines the consensus network; the values are: 0.4, 0.6, and 0.8. For each method and for a given threshold, we first quantify the number of edges that occur in at least one of the five runs, denoted as *T*. *T *is the cardinality of the superset of consensus edges derived from the union of the individual sets corresponding to the five runs. We plot count(*x*)/*T *for values of *x *= {1, 2, 3, 4, 5}, where count(5) indicates that an edge is present in the consensus networks in each of the 5 MCMC runs. At the highest threshold of 0.8, each of the three methods showed robustness to where at least 80% of the total edges are present in the consensus networks for all 5 MCMC runs. These findings are consistent with those of Zhu *et al. *[18]. Finally, Additional file 1 figure *S*1*d *depicts the number of edges that are recalled in all five MCMC runs at the three thresholds. The results suggest that our method offers relatively higher consistency across sampled networks.

### Possible Explanations for Increased Performance

There are two likely reasons for the enhanced performance associated with our SCT method. The first and most obvious reason is the increased coverage associated with the SCT method. The second reason is attributable to better resolution of ordered triplets. For example, consider the hypothetical sequential triplet influenced by a single locus: *L*_1 _→ *t_i _*→ *t_j _*→ *t_k_*. The LCMS procedure will generally establish the following relationships: *t_i _*→ *t_j_*, *t_i _*→ *t_k _*and *t_j _*→ *t_k_*. One practical limitation of this approach is that it does not usually prefer the motif *t_j _*→ *t_k _*over *t_i _*→ *t_k_*. That is, both pairwise orderings are likely with respect to the locus *L*_1_. In contrast, our method does not rely on a fixed anchor (genomic locus), and is capable of discriminating between potentially confounding motifs of this nature. This is due to the fact that, while both transitions of *t_i _*→ *t_j _*and *t_i _*→ *t_k _*are likely for our SCT method, the best scoring alignment corresponds to the true configuration, which will often be realized as the average configuration over multiple runs. In general, finding the optimal configuration will be self-perpetuating when various ordered triplets are interconnected, as the learning of causal relationships at lower rungs is clearly dependent on correct alignments in the upper levels.

## Discussion

We presented methodology aimed at utilizing genotypic data for the task of gene network reconstruction on eQTL datasets. Our method is motivated by previous studies focused on the same goal, however, we are able to provide improvements in coverage and resolution. Furthermore, with enhanced network reconstruction accuracy, we show that sampling a set of networks is efficacious at eQTL mapping. Although we followed established protocols for simulating eQTL data, it's inevitable that the simulated data does not perfectly model natural eQTL data. For example, our model omits feedback loops, though such motifs are common in real gene networks. Furthermore, we clearly are unable to model cases where genetic variations are associated with amino acid substitutions without corresponding expression changes. Other situations that we are unable to model include post-translational modifications, such as protein-phosphorylations and other mechanisms affecting protein concentrations. It is worth noting that, since our model is generally more complicated than univariate mapping techniques, it stands to reason that univariate mapping might be less sensitive to discrepancies between the model used in our study and real eQTL networks.

Future work involves applying our methodology to datasets that incorporate macroscopic phenotypes, including medical conditions and responses to pharmacological treatments [27]. Reconciling variations in macroscopic phenotypes with genotypic and expression variations is an emerging problem that is poised to yield great insights into the molecular bases of complex phenotypes. Already methods exist that focus on predicting the outcome of pharmacological treatments in the yeast eQTL dataset [28,29]. While the prediction of such outcomes are useful, we posit that accurate classification of an outcome is better viewed as a constraint within the general problem of learning a causal network. This formulation is more holistic and requires that learned networks agree with the classification of the macroscopic phenotype. Given that complex macroscopic phenotypes are often connected to several loci at relatively weak levels, our SCT method offers potential for this class of problems due to its ability to causally connect distal nodes in a network. Other possible applications include association studies, which carry an added degree of difficulty due to the genotypic heterogeneity associated with population-wide samples.

There are several possible ways in which our approach can be optimized. For example, we plan to investigate the use of iterative procedures, where information from prior runs is incorporated into subsequent runs to improve accuracy. With respect to the general area of Bayesian network structure learning, it would be interesting to consider integrating the causal ordering information from our method with other sources of prior biological information, such as protein-protein interactions or gene ontology (GO) annotation [30]. Such lines of inquiry should be feasible in light of the recent development of principled ways of incorporating prior information [31-33]. Finally, we note that our method utilizes parameters that are optimized a priori to structure learning. While this straightforward approach is able to provide appreciable performance improvements, further benefits might be realized by optimizing the parameterization of our method jointly with the beta parameter in the structure learning procedure; methodology addressing joint optimization problems in the context of Bayesian network structure learning was recently presented by Werhli *et al. *[33].

## Conclusions

We developed a probabilistic method based on stochastic causal trees to learn the causal relationships between gene transcripts in genetical genomics studies. Incorporating the information from our method as a prior into Bayesian network structure learning increases the performance of network reconstruction and eQTL mapping.

## Methods

### Estimating Network Properties

The synthetic network consists of 2, 200 transcripts and 50 loci, connected by 2, 598 edges. These values were chosen based on analysis run on the yeast eQTL data published by Kruglyak and colleagues [17]. We estimated the number of transcripts that show significantly variable expression patterns in the yeast eQTL dataset by considering the following criteria: 1) minimum variance of 0.06, 2) minimum correlation of 0.35 between the two fluorescent probes, and 3) minimum heritable variation of 0.5, where we measure heritable variation with the measure presented by Brem *et al. *[17]. As previously mentioned, we also chose to model 50 loci. This estimation is derived from assessing the number of loci conducive to 4 or more transcripts at the significance level of 1.0 × 10^-5 ^or lower. Next, correlated and adjacent loci were aggregated, yielding 50 genomic "epicenters." This number is roughly consistent with the two recent mapping studies of Zhang *et al. *[26] and Litvin *et al. *[24], which identified 25 and 44 loci, respectively.

### Network Simulation

**Figure 13:**
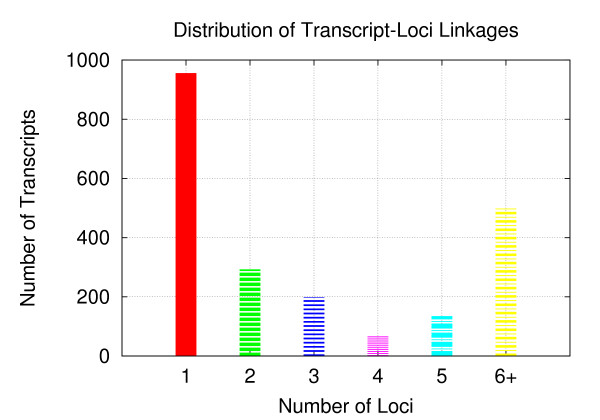
**Transcript-Loci Distribution**. The distribution of transcript-loci linkages. 60% of the transcripts link to 2 or more loci.

Given the established number of loci and transcripts, we next implemented a network-generating procedure that yields a level of complexity on par with real eQTL data in terms of the distribution of transcript-loci linkages. Step 1 involves randomly assigning the leaves to one of the 50 loci, where the assignment of the transcript can be to any of the nodes on the growing tree. At this point, every transcript is part of a tree rooted by a single locus, and the loci generally do not contain an equal number transcripts due to the random allocation of leaves. Step 2 involves randomly adding feed-forward edges and inter-loci edges. A feed-forward edge connects a transcript belonging to a particular locus to another transcript already belonging to that locus, whereas inter-loci edges connect transcripts that belong to different loci. The target ratio of inter-loci edges to feed-forward edges is 9 : 1, achieved by randomly selecting a number from a uniform distribution with a 0[1] interval, then deeming the operation as inter-loci if the number is < 0.9. Ultimately, at least 60% of transcripts have linkages to 2 or more loci, consistent with recent evidence indicating that many transcripts exhibit complex genetic bases [17]. The distribution of transcript-locus linkages for our synthetic network can be seen in Figure 13.

### Simulating eQTL Data

From the simulated network of 2, 200 transcripts and 50 loci, eQTL data are subsequently generated according to the protocol presented by Zhu *et al. *[18]. Genotypic data were randomly assigned values for either of the parental strains; genomic loci were assumed to be independent.

Expression traits were simulated according to a linear model:

$$y=\sum_{i}a_{i}x_{i}+\epsilon_{i}$$

The coefficient *a_i _*is drawn from a Gaussian distribution with a mean of 0.75 and standard deviation of 0.2. The error term is drawn from a Gaussian distribution with a mean of 0 and standard deviation of 1. All transcripts will have one or more parents (denoted by *x_i_*), and the parents may be of any composition of loci and transcripts. The sign of *a_i _*is assigned by drawing from a uniform distribution where the probabilities of positive and negative signs are equal to 0.7 and 0.3, respectively. A fraction (10%) of the transcripts with 2+ parents are selected at random to have non-linear interactions, and are modeled with an additional interaction term: $y=\sum_{i}^{N}a_{i}x_{i}+\sum_{i,j=1}^{N}k_{i,j}x_{i}x_{j}+\epsilon_{i}$

For these traits, the interaction term, *k*_*i*,*j*_, is drawn from a Gaussian distribution with a mean of 0.5 and a standard deviation of 0.1. Ultimately, the mean correlation between parent and child is 0.68. To generate eQTL data composed of weaker correlation structure, we drew *a_i _*from a Gaussian distribution with a mean of 0.6 and a standard deviation of 0.2. The network composed of weaker linkages has a mean correlation between parent and child of 0.55. Table 1 summarizes the properties of the eQTL datasets (rows 2-4), and the parameters used to generate the data (rows 5-8).

In summary, we generated both strongly and weakly correlated datasets, each composed of 100, 200, and 300 samples, resulting in six total datasets.

### Traditional Univariate eQTL Mapping

We used the t-test statistic to implement traditional univariate eQTL mapping, which involves an exhaustive search between all transcripts and loci. For each transcript-locus test, the expression levels for the transcript across all segregants are partitioned by the genotypes at the locus. Subsequently, the t-test is performed to assess the extent to which a locus influences the expression level of a transcript. This is repeated for all transcripts against all loci. In order to account for multiple hypothesis testing, we applied the false discovery rate (FDR) test of Benjamini *et al. *[34]. For eQTL mapping, this entails permuting the expression data prior to measuring the t-test statistic [35]. This shuffling procedure is repeated 10, 000 times to simulate the null distribution, and the null statistics are pooled across transcripts in order to obtain the "experimentwise" threshold [35]. Given this information, the FDR for a nominal p-value can be expressed as: $\text{FDR}=\frac{\text{false}\;\;\text{linkages}}{\text{true}\;\;\text{linkages}}$, where false linkages are defined as instances where the null hypothesis is falsely rejected.

### 0.1 LCMS Method

We implemented the LCMS method from Schadt and colleagues as outlined in their previous publications [12,13,18]. The LCMS method models triplets consisting of two transcripts and a genomic locus, with the purpose of resolving the true causal relationship between the two transcripts with respect to the genomic locus. The triplet consists of two transcripts, *t_x _*and *t_y_*, and one locus, *L_j_*, and can be resolved by any one of three models:

$$\begin{array}{l} M1=p(t_{x}\to t_{y}|t_{x},t_{y},L_{j})\\ M2=p(t_{y}\to t_{x}|t_{x},t_{y},L_{j})\\ M3=p(t_{x}╨t_{y}|t_{x},t_{y},L_{j}) \end{array}$$

Models *M*1 and *M*2 indicate a causal orientation, whereas *M*3 is indicative of an independent relationship. The likelihood of the models can be expressed as follows:

$$\begin{array}{l} M1=\prod_{i=1}^{N}\sum_{j=1}^{2}P(L_{j})l(\theta_{t_{x_{i}}|L_{j}};t_{x_{i}}|L_{j})l(\theta_{t_{y_{i}}|t_{x_{i}}};t_{y_{i}}|t_{x_{i}})\\ M2=\prod_{i=1}^{N}\sum_{j=1}^{2}P(L_{j})l(\theta_{t_{y_{i}}|L_{j}};t_{y_{i}}|L_{j})l(\theta_{t_{x_{i}}|t_{y_{i}}};t_{x_{i}}|t_{y_{i}})\\ M3=\prod_{i=1}^{N}\sum_{j=1}^{2}P(L_{j})l(\theta_{t_{x_{i}}|L_{j}};t_{x_{i}}|L_{j})l(\theta_{t_{y_{i}}|t_{x_{i}},L_{j}};t_{y_{i}}|t_{x_{i}},\;L_{j}) \end{array}$$

Each model applies to a locus, *L_j_*, and two transcripts, *t_x _*and *t_y_*. Each likelihood function iterates over the *N *possible experiments and the 2 possible states of *L_j_*. Likelihood functions of the form $l(\theta_{t_{x_{i}}|L_{j}};t_{x_{i}}|L_{j})$ are implemented as a bivariate normal distribution, whereas the likelihood function in M3, $l(\theta_{t_{y_{i}}|t_{x_{i}},L_{j}};t_{y_{i}}|t_{x_{i}},L_{j})$, is modeled as a conditional bivariate normal distribution. The complete functions are outlined in the supplemental material section of Schadt *et al. *[12].

Bootstrapping is applied 1, 000 times for each triplet, from which the probability of each of the respective models is obtained. Given these probabilities, the actual transcript-transcript priors are obtained by the following rules:

**If: **(*p*(*t_x _*╨*t_y|_t_x_*, *t_y_*, *L_j_*) > 0.5, then:

$$p(t_{x}\to t_{y})=1-\sum_{j}\frac{p(t_{x}╨t_{y}|t_{x},t_{y},L_{j})}{1}$$

**Else if: ***p*(*t_x _***→ ***t*_*y*_|*t*_*x*_, *t*_*x*_, *L*_*j*_) > 0.5, then:

p(tx→ty)=∑j2∗p(tx→ty|tx,ty,Lj)p(tx→ty|tx,ty,Lj)+p(ty→tx|tx,ty,Lj)

To summarize this logic, the authors prefer to downweight the prior score over two transcripts in cases where the independent model has a probability greater than 0.5.

### Stochastic Causal Tree Method

The stochastic causal tree method is a probabilistic procedure for learning causal hierarchies representing the propagation of influence that emanates from genomic loci and is transmitted through gene transcripts. The trees consist of genomic loci, which serve as roots for their respective trees, and an arbitrary number of transcripts that are stochastically added to the growing tree. The integrity of the branches are maintained with a combination of second- and third-order potentials that act in concert to maintain causal alignments. Once the tree is initiated with a particular locus serving as a root, the crux of the method involves choosing optimal transitions, assessed by the likelihoods associated with transcripts being added as leaves to the growing tree. We express the likelihood of a transition as the sum of the likelihoods of two potentials involving the grandparent (*n_g_*), parent (*n_p_*), and child (*n_c_*) nodes. The potential functions can be expressed abstractly as:

(1) $$\phi (n_{p},n_{c})$$

(2) $$\phi (n_{g},n_{c}|n_{p})$$

There are several functions that could reasonably be used to implement the potentials, including Pearson's correlation, mutual information, or regression functions. We considered both the PCC and regression functions for our study. Ultimately, due to the fact that eQTL datasets consist of both binary (loci) and continuous (expression) data, we opted to employ linear regression functions to model the potentials. In addition to being suitable for modeling datasets composed of binary and continuous variables, regression functions lend adaptability to studies involving diploids where heterozygosity can be represented by a separate category from either homozygous state. Thus, all figures corresponding to our SCT method in this manuscript are derived from an implementation with regression functions. However, for reference we include a performance comparison between the PCC and regression functions in Additional file 1, figure *S*1. We found that both approaches yield comparable results. For the second-order potential, *ϕ*(*n_p_*, *n_c_*), we use the following function:

$\phi (n_{p},\;n_{c})=\sqrt{SSR_{n_{p},n_{c}}/SSTO_{n_{p},n_{c}}}=\sqrt{R_{n_{p},n_{c}}^{2}}$, where $SSR_{n_{p},n_{c}}$ and $SSTO_{n_{p},n_{c}}$ are the regression sum of squares and the total sum of squares, respectively, from the linear regression model: ***n*_c _**= *β*_0 _+ *β*_1 _* **n_p _**+ ϵ For the third-order potential, *ϕ*(*n_g_*, *n_c_|n_p_*), we employ a two-step regression procedure:

**1**. Let e be the residuals from the linear regression model: n_c _= *β*_0 _+ *β*_1 _* n_p _+ *ϵ*

**2**. Set $\phi (n_{g},n_{c}|n_{p})=\sqrt{SSR_{n_{g},e}/SSTO_{n_{g},e}}=\sqrt{R_{n_{g},e}^{2}}$, where $SSR_{n_{g},e}$ and $SSTO_{n_{g},e}$ are the regression sum of squares and total sum of squares, respectively, from the linear regression model: **e **= *β*_0 _+ *β*_1 _* **n_g _**+ *ε *If *n_p _*is an intermediate between *n_g _*and *n_c_*, then the residuals from the first step will not result in a high value for $\phi (n_{g},n_{c}|n_{p})=\sqrt{SSR_{n_{g},e}/SSTO_{n_{g},e}}=\sqrt{R_{n_{g},e}^{2}}$ from the second linear regression function. In other words, *n_g _*will not be able to predict anything significant about *n_c_*, given that *n_c _*has already been regressed against *n_p_*. We use the ordinary least squares (OLS) method to estimate the beta parameters [36].

Given concrete functions to implement the second- and third-order potentials represented by equations 1 and 2, we wish to model the likelihood of obtaining any value for the potentials:

(3) $$l(\phi (n_{p},n_{c}))$$

(4) $$l(\phi (n_{g},n_{c}|n_{p}))$$

*l*(*ϕ*(*n_p_*, *n_c_*)) assesses the likelihood of obtaining a value for *ϕ*(*n_p_*, *n_c_*), and depends on the sampling distributions for nodes *n_p _*and *n_c_*. Modeling of the sampling distributions allows one to account for the fact that nodes vary in the extent to which they interact with other nodes, and this factor will influence the likelihood of obtaining a value for *ϕ*(*n_p_*, *n_c_*). To this end, we use the bivariate Gaussian function to approximate the distribution of interaction strengths, a technique that Faith *et al. *have demonstrated to be effective for modeling gene interactions in microarray data [37]. For each node *n_p_*, we employ a random sampling procedure to measure the node's "background" mean and standard deviation, denoted as $\mu_{\phi (n_{p},.)}$ and $\sigma_{\phi (n_{p},.)}$, respectively. The sampling values for each node *n_p _*are obtained by randomly choosing interaction partners while holding *n_p _*fixed. Given the sampling means and standard deviations for nodes *n_p _*and *n_c_*, *l*(*ϕ*(*n_p_*, *n_c_*)) is calculated as

$$\begin{array}{c} l(n_{p},\;n_{c})=\frac{1}{Z}\exp\left(-\frac{\nu_{n_{p}}^{2}-2\rho \nu_{n_{c}}\nu_{n_{p}}+\nu_{n_{c}}^{2}}{2(1-\rho^{2})}\right)\\ Z=2\pi \sigma_{\phi (n_{p},.)}\sigma_{\phi (.,n_{c})}\sqrt{1-\rho },\\ \nu_{n_{p}}=\frac{\phi (n_{p},n_{c})-\mu_{\phi (n_{p},.)}}{\sigma_{\phi (n_{p},.)}},\\ \nu_{n_{c}}=\frac{\phi (n_{p},n_{c})-\mu_{\phi (.,n_{c})}}{\sigma_{\phi (.,n_{c})}} \end{array}$$

The covariance, *ρ*, is assumed to be zero in our study. Also, genomic loci are restricted to the explanatory variable when obtaining the sampling distributions. Note that for two continuous variables, the $\sqrt{R^{2}}$ value is symmetric and equal to the correlation coefficient.

For the conditional potential, *ϕ*(*n_g_*, *n_c_|n_p_*), we assume a mean of 0.0. To estimate the standard deviation, we apply a sampling procedure whereby data for linear triplets are randomly generated and the conditional potential is computed. Additional file 1 table S2 contains the standard deviations used in our study and a description of the sampling process and linear models used to estimate the values. We denote the estimated standard deviation for all triplets as. $\sigma_{\phi (n_{*},n_{*}|n_{*})}$. Finally, the value of *l*(*ϕ*(*n_g_*, *n_c_|n_p_*)) is obtained from the following univariate normal probability density function:

l(ng, nc|np)=12πσϕ(n*,n*|n*)2exp(−ϕ(ng,nc|np)22σϕ(n*,n*|n*)2)

Equations 3 and 4 are used in tandem to model the likelihood that a transcript should be included as a leaf on a growing tree, and the log likelihood score is expressed as follows:

(5) LLS(nc, np, ng)=c1*log(l(ϕ(ng, nc|np)))−log(l(ϕ(np, nc)))

where *c_1 _*is a constant used to modulate the extent to which the conditional potential is weighted against the pairwise potential. We discuss the optimization of this constant in the "Parameter Optimization" subsection. Finally, we note that equation 5 represents a likelihood model and is not a formal decomposition of the joint probability, in which case the probability of the causal chain would be represented by (*n_g_*) * (*n_p_|n_g_*) (*n_c_*|*n_p_*).

**Figure 14:**
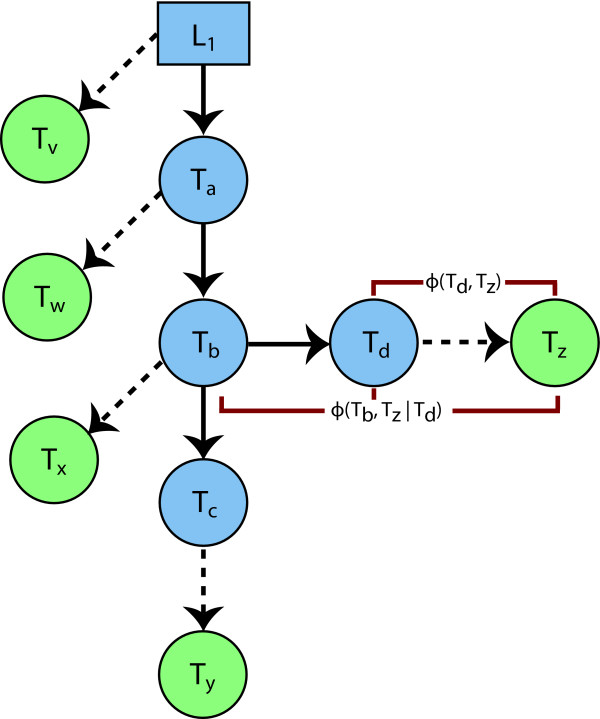
**Stochastic Causal Tree Schematic**. Schematic of the stochastic causal tree method. Blue nodes represent the locus (square) and transcripts (circles) that are currently part of the tree, with causal edges denoted by solid arrows. Optimal transitions for the locus and the four transcripts, *T_a_*, *T_b_*, *T_c _*and *T_d_*, are represented by dashed arrows to their respective target transcripts (green circles). The likelihood of any candidate transcript being added to the tree depends on the candidate, its parent, and its grandparent. For example, the likelihood of the candidate transcript *T_z _*being connected to *T_d _*is a function of two potentials: *ϕ*(*T_d_*, *T_z_*) and *ϕ*(*T_b_*, *T_z_*|*T*_*d*_). The potentials for this particular move are depicted in red.)

To describe the SCT method conceptually, the starting points of the algorithm are at the genomic loci, each of which serve as a root for their respective trees. As an example, we refer to a hypothetical tree depicted in Figure 14. The current state of the tree includes one locus, *L*_1_, and four transcripts, *T_a_*, *T_b_*, *T_c _*and *T_d_*. There are five potential target leaves corresponding to the optimal transitions for the locus and the four transcripts, where an optimal transition involves adding an unincorporated transcript as a leaf.

The SCT algorithm will stochastically choose between the five optimal transitions. The choice is weighted by a factor of the log likelihood score to a power represented by parameter *p*. Higher values of *p *bias the stochastic choice towards higher log likelihood scores. We discuss the importance of this weighting in the "Parameter Optimization" subsection. This process is repeated until the maximum number of nodes allowed per tree is reached, represented by the parameter *M*. We set *M *= 60, making it likely that the trees cover a number of transcripts equal to the ratio of transcripts to loci. We discuss how performance is affected by varying values of *M *in the "Parameter Optimization" subsection. Finally, we obtain *ϕ*(*n_g_*, *n_c_|n_p_*) for the root nodes by randomly generating a value according to a Gaussian distribution with parameters *μ *equal to 0.0 and *σ *equal to the value obtained via the sampling procedure described in Additional file 1 table S1. In practice, we found that simulating random values for the conditional potential is as effective as optimizing a fixed parameter.

The SCT method produces a set of trees which can be represented as adjacency matrices. The SCT output is converted into an *n *× *n *prior matrix by dividing the frequency at which node *x *is a parent of node *y *by the number of times the SCT method is run. For example, we ran the method 1, 000 times from each of the 50 loci. If node *x *is a parent of node *y *on 100 occasions, then the prior matrix will have a value of $\frac{100}{50,000}$ entry *x*, *y*, since there are 50 * 1, 000 trees constructed.

Formally, the SCT method is described as follows:

The dataset *D *is composed of two components, gene expression and genotypic data. We represent the expression data *E *as the set of *N *variables (genes) *x_i_*, ... *x_N _*∈ *X*. Similarly, the genotypic dataset *G *consists of the set of M variables (loci) *L_i_*,...*L_M _*∈ *G*. There are *P *samples (segregants), and therefore the two components of the dataset *D *can be expressed as: *E *= **x**[*h*]....**x**[*P*] and *G *= **g**[*h*]....**g**[*P*]. These data represent the input to the algorithm.

**Input **: The dataset, *D *= {*d*[1], ..., *d*[*P*]}

**Input **: *M*, the maximum number of leaves on a tree

**Input **: *I*, the number of iterations for the procedure (conducted over all loci)

**Input **: *L*, the set of *l *loci

**Output**: *R*, the *N *× *N *prior matrix representing the causal relationships between nodes

**for ***i *← 1 to *I ***do**

   **for ***l *∈ *L *do

      S←∅;// S is the set nodes included in the tree

      include node *l *into the set *S*;

      **for ***m *← 1 to *M *do

         *leaf *← StochasticLeaf(*S*);

         *S *← *S*∪ {leaf}; // add leaf to the set, *S*

         *m *← *m *+ 1;

   *i *←*i *+ 1

// Convert the trees into the prior matrix, *R*.

$\sum_{i,j}^{N}R_{i,j}=\frac{f(i,j)}{M*|L|*I}$, where *f*(*i*, *j*) represents the number of directed edges from node *i *to node *j*, summed over all trees.

**Algorithm 1**: SCT Main Procedure

**Input **: *S*, the set of nodes currently included in the tree

**Output**: the next leaf to be included in the tree

// *B *is the set of best candidates

*B←∅*;

**for **s **∈ **S **do**

   *b *= GetBestTransition(*s*.*id*, *s. parentId*);

   *B *← *B*∪{*b*};

return *b *∈ *B*, where *b *is stochastically chosen based on relative scores.

**Algorithm 2**: StochasticLeaf (Node[] S)

**Input **: *p*, the leaf node

**Input **: *g*, the parent of *p*

**Output**: the optimal transition corresponding to *p *and *g*;

// *C *is the set of candidates for node *p*

**for ***c ***∈ ***C ***do**

   *score*_*g*,*p*,*c *_= *c*_1_*log*(*l*(*ϕ*(*g*, *c|p*))) - *log*(*l*(*ϕ*(*p*, *c*)))

return *c_best _*∈ *C*, where *c_best _*corresponds to max(*score*_*g*,*p*,*_);

**Algorithm 3**: GetBestTransition (int p, int g)

### Bayesian Network Structure Learning

Bayesian networks provide a graphical representation of the joint probability distribution for a set of random variables, allowing for efficient computation of the probability of graphical structures [19]. A signature aspect of Bayesian networks is that the global likelihood function that defines the probability of the structure is decomposable into the product of local likelihood functions. The local likelihood score for each node can be computed based on the sufficient statistics encoded by its parents. Formally, Bayes' formula allows the posterior probability of a network, *P*(*G|D*), to be calculated as *P*(*G*) * *P*(*D|G*). Since the number of possible network structures grows super-exponentially with the number of nodes, it is not possible to conduct an exhaustive search over the space of possible networks. In light of this practical limitation, we use Markov Chain Monte Carlo (MCMC) simulations to explore the space of high-scoring network structures [38] from which we sample to construct a consensus network.

To improve the computational feasibility of the structure learning algorithm, we restrict the maximum number of parents to be three, a constraint that is employed by several other studies [13,18,32,38,39]. We further confine the search space by only considering edges between a node and a predefined set of candidates for each node. To construct the candidate set, we initially calculate each node's top-k associations. We next traverse the top-k sets to ensure reciprocity. For example, if node *y *is a top-k candidate of node *x*, we establish node *x *as a candidate of node *y*, if it wasn't already. This symmetry ensures that the transition matrix is reversible. For this study, we chose *k *= 10, which results in roughly 15 candidates per node once reciprocity is considered (i.e. the candidate set is expanded). This relatively stringent candidate set is chosen for computational efficiency, but it does encompass nearly 97% of the true edges for each of the six datasets.

MCMC simulations are initialized with a graph consisting of 2, 000 randomly selected edges, after which the evolution of the structure is governed by the acceptance function. Formally, the acceptance probability is expressed as follows:

$$A(G^{\prime }|G)=min\left\{\frac{P(D|G^{\prime })P(G^{\prime })Q(G;G^{\prime })}{P(D|G)P(G)Q(G^{\prime };G)},\;1\right\}$$

New structures are drawn from the proposal distribution, *Q*(*G*; *G'*), and the proposed structure *G' *is randomly chosen from the set of legal moves corresponding to *G*, which is denoted as *η*(*G*). *η*(*G*) is constrained by the following criteria: 1) the move involves the addition, removal, or reversal of an existing edge; 2) the move does not result in a cycle; 3) the move does not result in any node having more than three parents; and 4) the move involves a candidate node. One subtle aspect of the proposal distribution is that is not necessarily symmetric [32,38]. Therefore, *η*(*G*) and *η*(*G'*) must be explicitly calculated and accounted for in the acceptance function. The computation of the Hastings factor, *Q*(*G*; *G'*)/*Q*(*G'*; *G*), thus reduces to the ratio of cardinalities of the two sets: $Q(G;G^{\prime })/Q(G^{\prime };G)=\frac{\left|\eta (G)\right|}{\left|\eta (G^{\prime })\right|}$. We note that maintaining *η*(*G*) is made computationally feasible by dynamically tracking node ancestries during the simulations.

Following recent research on the subject of incorporating prior biological knowledge into the Bayesian network structure learning procedure [31-33,40], we model information corresponding to our SCT method and the LCMS method with the Gibbs distribution. Imoto *et al. *[31] formulate the prior over the distribution of possible structures as:

$$P(G|\beta )=\frac{e^{-\beta E(G)}}{Z(\beta )}$$

where *E*(*G*) is the energy function, and the partition function *Z*(*β*) is the normalizing constant. The hyperparameter *β *represents the inverse temperature and can be used to modulate the strength of the prior information as compared to the Bayesian network likelihood score. Exact computation of *Z*(*β*) entails enumerating over all possible network structures, which is not computationally feasible. An upper bound estimate for the partition function *Z*(*β*) has been presented by others [31,41]; however, since the structure learning procedure evaluates the ratio of two likelihood functions, the normalizing constant cancels out in the acceptance function, leaving a ratio of two exponentials. Let R equal the *n × n *matrix representing prior information from the LCMS or SCT methods. The calculation of the graph prior, P(G), is as follows:

$$P(G)=\exp\{\beta \sum_{i,j\in e(G)}R_{i,j}\}$$

Where *e*(*G*) denotes the set of edges in *G*.

The edge frequencies over a set of sampled graphs {*G*_1..._*G_N _*} can be calculated via model selection: $f(e(G))=\sum_{n=1}^{N}I(e(G_{n}))*P(G_{n}|D)$, where *I*(*e*(*G_n_*)) indicates the presence of individual edges in the *n_th _*sampled network. For our study, we employ a simulation-consistent approximation of the posterior edge frequencies [42]:

$$\begin{array}{c} E[f(e(G))]=\frac{1}{N}\sum_{n=1}^{N}I(e(G_{n}))\\ ≃\sum_{n=1}^{N}I(e(G_{n}))*P(G_{n}|D) \end{array}$$

For each of the three structure learning methods, and for each dataset, we initiate two parallel MCMC runs. Each run consists of 150 million iterations with a burn-in period of 10 million iterations. We assess convergence in the subsequent 140 million iterations. As has been noted by others [38,39,41], a necessary but not sufficient property for convergence is the apparent stabilization of edge frequencies. Thus a simple heuristic to assess convergence involves measuring the distortion between sampling intervals, defined as *S_i_*, where *i *indicates the *i^th ^*interval. We sample a network at every 200 iterations, leading to 140M/200 = 700, 000 total networks sampled in each run. Upon convergence, the distortion in edge frequencies between sampling intervals should converge, both within and between MCMC simulations. Let **S_A _**be the set of sampling intervals for the first MCMC simulation, and let **S_B _**be the set for the second MCMC simulation. Upon convergence, the intra-run distortions, *D*(*S*_*A*,*i*_, *S*_*A,i*+1_) and *D*(*S*_*B,i*_, *S*_*B*,*i*+1_), should converge to the same level as the inter-run distortions, *D*(*S_A,i_*, *S*_*B*,*i*_). We define distortion as the difference in edge frequencies over a sampling interval. For example, if an interval consists of 100 samples, and if edge *i*, *j *occurs in $\frac{90}{100}$ samples for interval *A *and in $\frac{70}{100}$ samples during interval *B*, then the distortion for edge *i*, *j *is 0.9 - 0.7 = 0.2. To obtain the distortion for a graph, we average over the distortions of all possible edges. We found that 150*M *iterations was sufficient to demonstrate that the distortion between the two MCMC runs is reduced to 0.005. Additional file 1 figure *S*2 illustrates convergence for all three methods when applied to the dataset with weaker correlation structure and 200 samples. Specifically, figure *S*2 shows the relationship between performance (AUC) and convergence (distortion) as the number of iterations increases. It is clear that performance (AUC) reaches a plateau after 20*M *iterations (log(20*M*) = 7.3), indicating that, although edge distortion will in theory reach 0.0 over an infinite number of iterations, optimal performance is attained within 150*M *iterations. Another useful way to illustrate convergence is to plot the edge frequencies from two independent runs. Additional file 1, figures *S*3*a*-*S*3*c*, corresponding to each of the respective methods, indicate strong concurrences in edge frequencies between runs for all three methods.

Optimal values of *β *for both the LCMS and SCT methods are determined by measuring the performance of network reconstruction over a broad range of values in the following set of integers: {4, 8, 12, 16, 20, 24}. Tables 2 and 3 show how different values for *β *affect performance on datasets with stronger and weaker correlations, respectively. Optimal values are shown in bold. Both the LCMS and SCT methods proved stable across different values of *β*.

**Table 2:** Beta Parameter; Stronger Correlation

Method	Beta	100 Samples	200 Samples	300 Samples
LCMS	4	0.628	**0.867**	**0.926**
LCMS	8	**0.646**	0.856	0.925
LCMS	12	0.645	0.854	0.920
LCMS	16	0.628	0.845	0.906
LCMS	20	0.616	0.831	0.894
LCMS	24	0.601	0.817	0.881

SCT	4	0.783	0.926	0.953
SCT	8	0.821	0.943	0.964
SCT	12	0.837	0.953	0.969
SCT	16	**0.843**	**0.959**	0.967
SCT	20	0.841	0.958	0.968
SCT	24	0.842	0.957	**0.969**

**Table 3:** Beta Parameter; Weaker Correlation

Method	Beta	100 Samples	200 Samples	300 Samples
LCMS	4	0.577	0.787	0.884
LCMS	8	0.578	**0.788**	0.884
LCMS	12	**0.587**	0.781	**0.886**
LCMS	16	0.574	0.786	0.885
LCMS	20	0.574	0.773	0.880
LCMS	24	0.562	0.773	0.881

SCT	4	0.689	0.847	0.925
SCT	8	0.715	0.869	0.935
SCT	12	0.726	0.878	0.939
SCT	16	**0.726**	0.883	0.940
SCT	20	0.724	**0.885**	**0.941**
SCT	24	0.725	0.884	0.939

The continuous data are discretized via k-means clustering into three levels representing down-regulated expression, steady expression, and up-regulated expression. The data are parameterized with a multinomial distribution and we utilized the structure-equivalent Dirichlet priors introduced by Heckerman *et al. *[43].

Therefore, while both the LCMS and SCT methods utilize continuous data to produce a prior matrix, the Bayesian network structure learning procedure uses discrete data. We preferred the computational efficiency associated with discrete data given the large number of simulations conducted in our study. Finally, we note that genomic loci are modeled as head variables in our networks, which has two consequences: 1) we do not need to test for cis- versus trans-relationships; 2) We are able to use the resulting networks for expression quantitative trait loci (eQTL) mapping.

### Parameter Optimization

The conditional weight parameter *c*_1 _and the power parameter *p *are optimized via a grid search. For datasets composed of 200 experiments, the results are depicted in Tables 4 and 5, corresponding to datasets with stronger and weaker correlation structure, respectively. The figures represent the area under precision-recall curves (AUC), where the performance is for network reconstruction by the SCT method alone, and not the SCT-augmented Bayesian networks. For both strongly and weakly correlated datasets, performance degrades considerably as the conditional weight parameter tends to 0.0. Furthermore, for both strongly and weakly correlated datasets, it is optimal to weight the power by a factor of 3.0 or 4.0. Similar results were obtained for datasets composed of 100 and 300 samples (data not published). In this study, we use *c*_1 _= 5.0 and *p *= 3.0.

**Table 4:** c1 and p Parameter Optimization; Stronger Correlation; 200 Samples

		p			
		**1.0**	**2.0**	**3.0**	**4.0**

**c_1_**	0.0	0.606	0.668	0.672	0.660
	1.0	0.661	0.740	0.751	0.751
	2.0	0.686	0.777	0.801	0.790
	3.0	0.718	0.804	0.824	0.812
	4.0	0.736	0.823	0.839	0.822
	5.0	0.753	0.836	0.850	0.857
	6.0	0.771	0.841	0.854	0.836

**Table 5:** c1 and p Parameter Optimization; Weaker Correlation; 200 Samples

		p			
		**1.0**	**2.0**	**3.0**	**4.0**

**c_1_**	0.0	0.600	0.652	0.643	0.635
	1.0	0.630	0.692	0.698	0.668
	2.0	0.662	0.722	0.726	0.700
	3.0	0.677	0.739	0.746	0.722
	4.0	0.698	0.772	0.762	0.738
	5.0	0.714	0.768	0.778	0.750
	6.0	0.727	0.798	0.786	0.782

**Figure 15:**
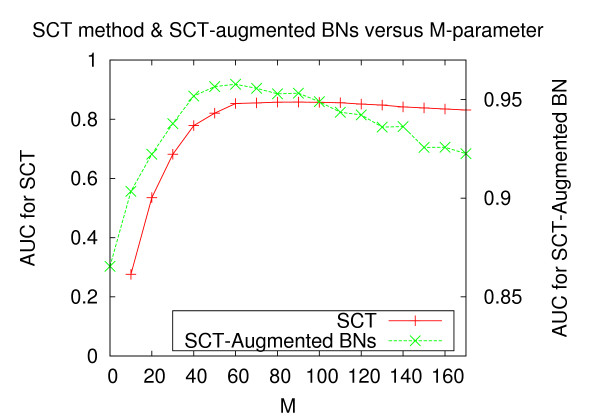
**M Parameter Optimization**. The performance of the SCT method alone (y1 axis) and the performance of the SCT-augmented Bayesian networks (y2 axis) versus the M-parameter (x axis). In both cases, AUC refers to the area under the precision-recall curves. *M *= {40, 100} represents a broad range over which our method offers notable performance improvements.

Parameter *M *represents the number of leaves that are modeled in each tree. Figure 15 depicts network reconstruction performance against a range of values for *M*. For the dataset composed of strongly correlated data and 200 samples, we plot the performance of network reconstruction for the SCT method alone (dashed, y1 axis), and for the SCT-augmented Bayesian networks (solid, y2 axis). We plot both side-by-side to assess the extent to which SCT performance acts as a proxy for SCT-augmented Bayesian networks when varying *M*. The AUC for unaugmented Bayesian networks is depicted on the *y*1 axis where *M *= 0 (i.e. no SCT influence). As *M *increases from 10 to 100, performance of the SCT and SCT-augmented Bayesian networks both increase. In this study, we used *M *= 60 for all reported figures. Performance for network reconstruction conducted by SCT-augmented Bayesian networks shows a gradual decline starting at *M *= 110, which mirrors the decline in the SCT method alone. This reflects the fact that higher values of *M *allow more chances for erroneous structures. In summary, there is a broad range of values over which our method is effective. If *F *is the ratio of total variables to loci, denoted as $F=\frac{\left|N\right|}{\left|L\right|}$, then this study suggests that values of *M *= {1.0*F*, 2.5*F*} would provide strong performance improvements. It is also easy to imagine additional stopping criteria that could improve performance further, such as a minimal log-likelihood score for leaf inclusion.

### Precision-recall curves

To assess the quality of the reconstructed networks, we generated precision-recall plots, which provide a graphical depiction of precision versus recall. Precision is defined as $\text{precision}=\frac{\neq \text{true}\;\text{positives}}{\neq \text{true}\;\text{positives}+\neq \text{false}\;\text{positives}}$, and recall is defined as $\text{recall}=\frac{\neq \text{true}\;\text{positives}}{\neq \text{total}\;\text{true}\;\text{positives}}$. Precision indicates the sensitivity of the method, whereas recall indicates the extent to which the method can detect true edges. Higher levels of precision and recall are both desirable, and thus so are higher values of area under the curve (AUC).

For causal edge detection, we construct precision-recall curves by sampling 1:4 M networks at every 200 iterations over an interval consisting of 140 M iterations for two individual MCMC runs. Therefore, the total number of networks sampled is expressed as: $2\;\text{runs}*\frac{140\;\text{M}\;\text{iterations}}{\text{run}}*\frac{1\;\text{network}}{200\;\text{iterations}}=1.4\text{ M}$ networks.

Though far fewer networks would likely be sufficient to assess performance, we note that at least 10, 000 thousand samples are ideal, given that any sampled network from the MCMC chain will contain many suboptimal edges. To generate precision-recall curves for the consensus network, we lower the threshold frequency at or above which an edge occurs, starting at 1.4 M. If an edge occurs at or above the threshold, it is assigned a true or false positive based on whether the edge is present in the true network. The threshold is repeatedly decremented by 1 until reaching 0.

For eQTL mapping, we take individual networks and establish transcript-loci (eQTL) linkages by conducting a depth-first from each locus. That is, an eQTL linkage is established if there is a directed path from a locus to a transcript. Since any sampled network from the MCMC chain will contain a considerable number of extraneous edges, we extract 1, 000 networks that are spaced 5, 000 iterations apart, then use the extracted networks as initializations for greedy optimization procedures [44] with 200, 000 iterations. This results in the removal of suboptimal edges that are deleterious to eQTL mapping. In summary, for each method and for each of the six datasets, we greedily optimized 1, 000 networks upon which we conducted depth-first searches to establish eQTL linkages. The depth-first searches on the individual networks yields 1, 000 sets of transcript-locus linkages. We generate precision-recall curves by lowering the threshold frequency at or above which a transcript-locus linkage occurs, starting at 1, 000 and decrementing by 1 until reaching 0. True eQTL linkages are established by running depth-first searches on the true network.

## Availability

Software for the SCT method and MCMC Bayesian structure learning procedure can be accessed online: http://www.cs.ucsb.edu/%7Edbl/software.php

## Authors' contributions

KC and AS designed the study. KC implemented the software and carried out the experiments. Both authors read and approved the final manuscript.

## Supplementary Material

Additional file 1 **Supplementary Figures**. Supplementary Figures 1a-1 d, 2, 3a-c; Supplementary Tables 1a-b, 2a-b. Click here for file
