# Supplementary material for: Using Stochastic Causal Trees to Augment Bayesian Networks for Modeling eQTL Datasets
Source: BMC Bioinformatics. 2011 Jan 6;12:7. doi: 10.1186/1471-2105-12-7 (PMC3032670; doi:10.1186/1471-2105-12-7)
Supplement: Additional file 1 — Supplementary Figures. Supplementary Figures 1a-1 d, 2, 3a-c; Supplementary Tables 1a-b, 2a-b. [file 1471-2105-12-7-S1.PDF]

Supplementary Figures 1a-1d.

1a)

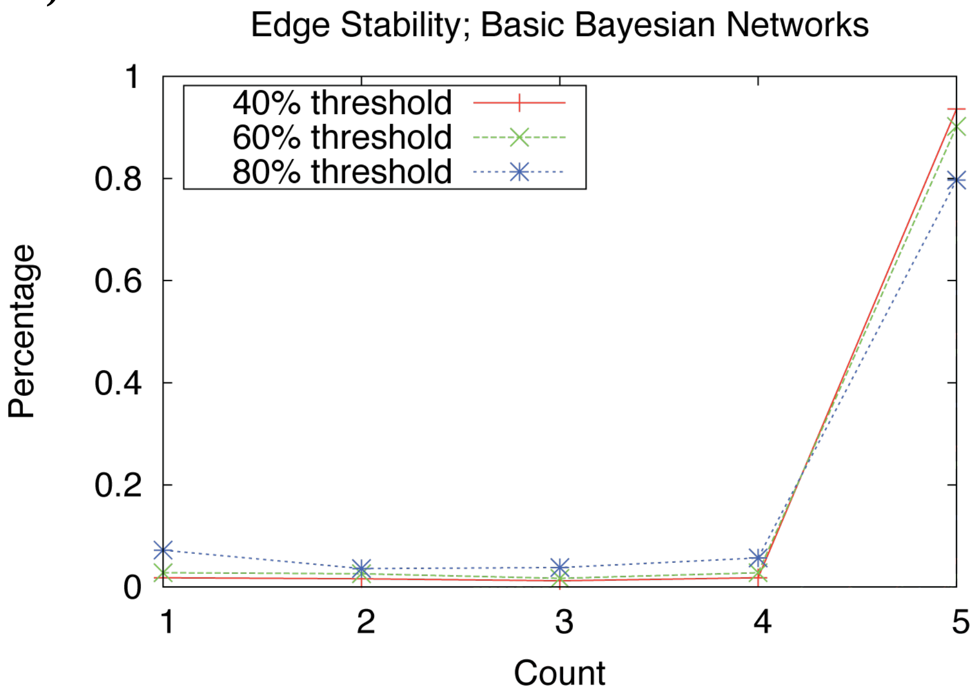

1b)

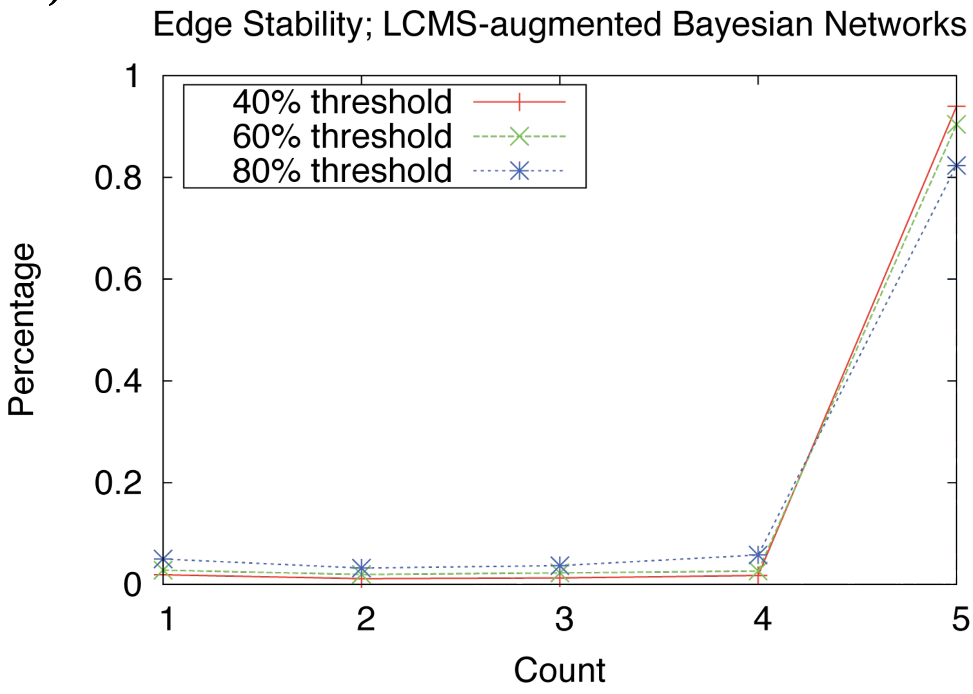

1c)

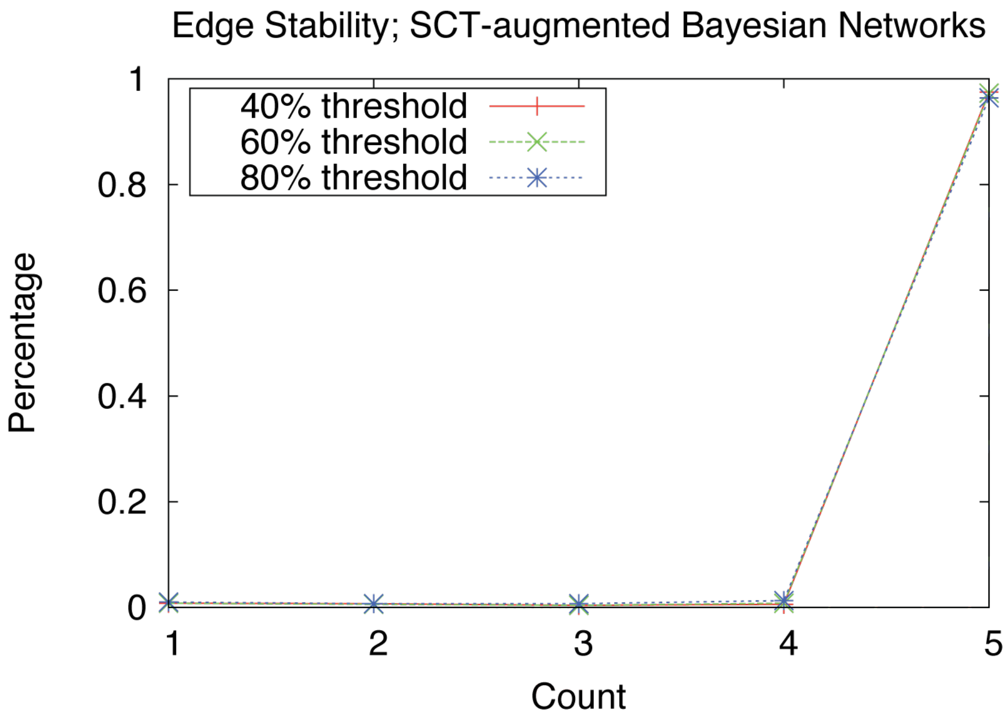

Supplementary figures 1a-1c depict network reconstruction stabilities for weaker correlations and 200 samples, presented for each of the three methods: (a) unaugmented, (b) LCMS-augmented, and (c) SCT-augmented Bayesian networks. For each method, we ran 5 separate MCMC runs of 150 million iterations. Networks were sampled after a burn-in period of 10 million iterations. For each method, we determine T, the total number of edges that are present in the consensus networks for at least 1 of the 5 MCMC runs. We adjusted the frequency threshold that determines the consensus network; the values are: 0.4, 0.6, and 0.8. We plot count(x)/T for values of x = {1,2,3,4,5}, where count(5) indicates that an edge is present in the consensus networks for all 5 MCMC runs.

1d)

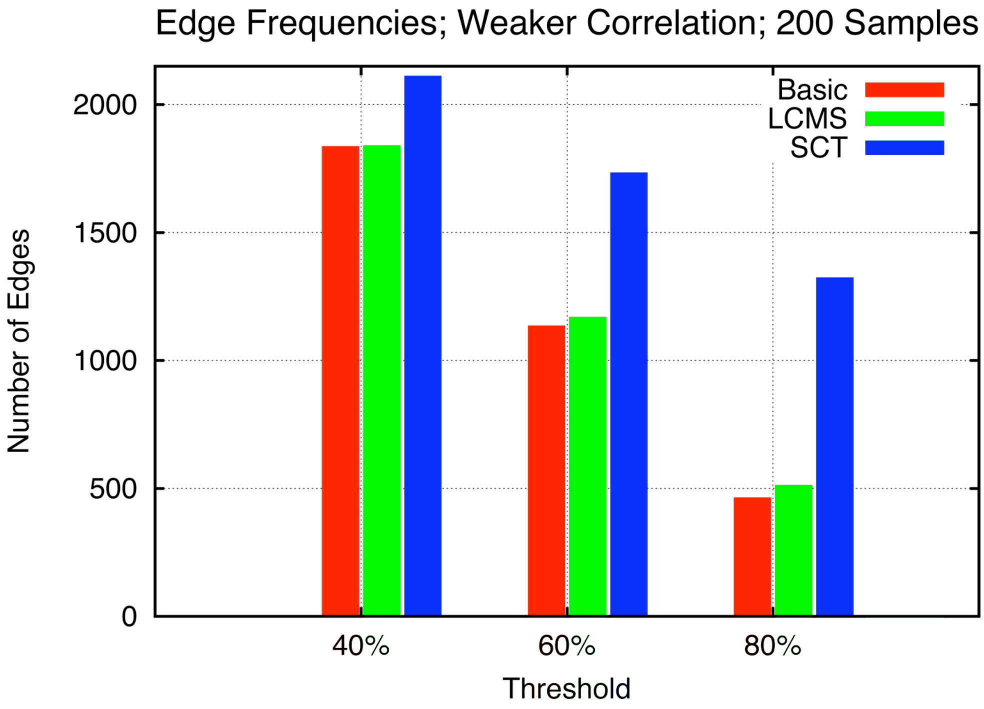

Supplementary figure 1d, which corresponds to supplementary figures 1a-1c, depicts the number of edges that are recalled in the consensus networks for each of the 5 MCMC runs at a certain threshold. For example, at the 80% threshold, SCT-augmented Bayesian networks recover 1,324 edges common to all 5 MCMC simulations, whereas unaugmented Bayesian networks only recall 464 edges.

Supplementary Figure 2.

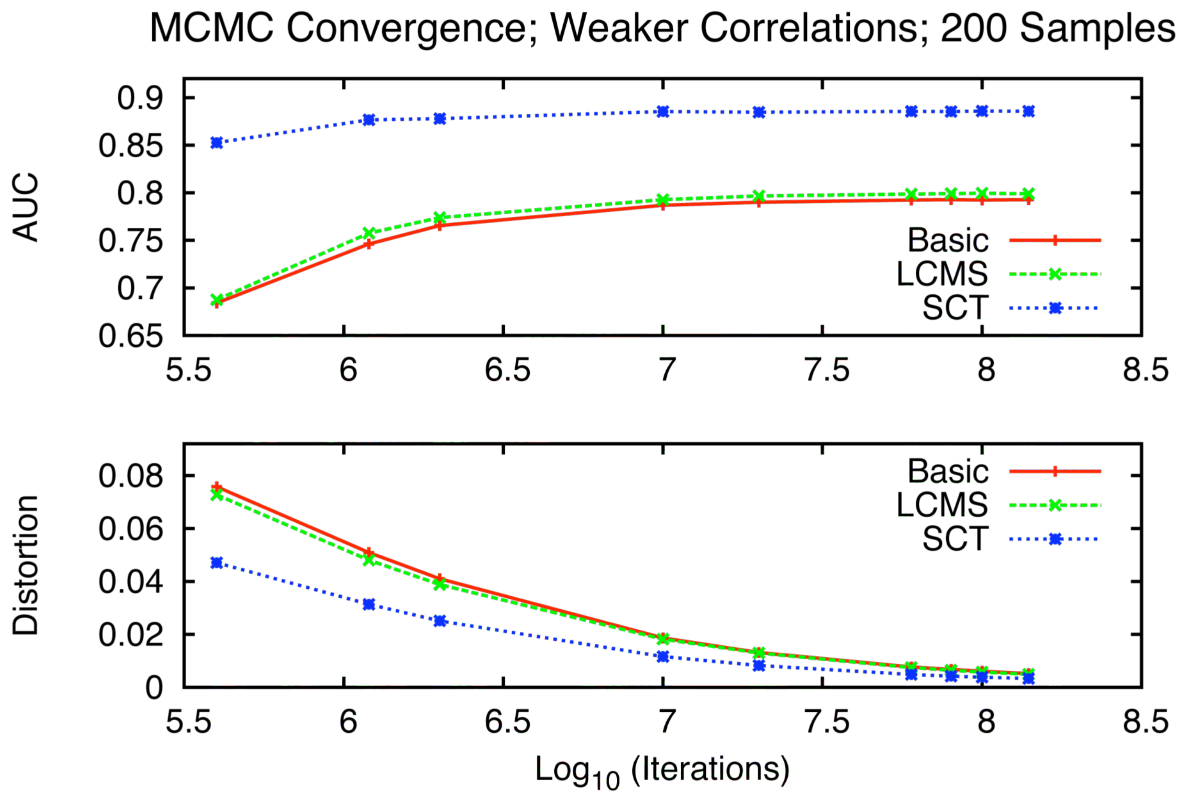

The progression of convergence and performance for all three methods when applied to the dataset with weaker correlation structure and 200 samples. This figure depicts performance (AUC, top graph) and convergence (distortion, bottom graph) versus the number of iterations (x axis). From the top graph, it is clear that the AUCs reach a plateau at roughly $\log_{10}$ (20 M) = 7.3 iterations. On the bottom graph, it is evident that the SCT-augmented networks converge more quickly, but all three methods descend to 0.005 after $\log_{10}$ (150 M) = 8.16 iterations. Taken together, we are confident that 150M iterations is sufficient to produce optimal network reconstruction performance for all three methods.

Supplementary Figures 3a-3c.

3a)

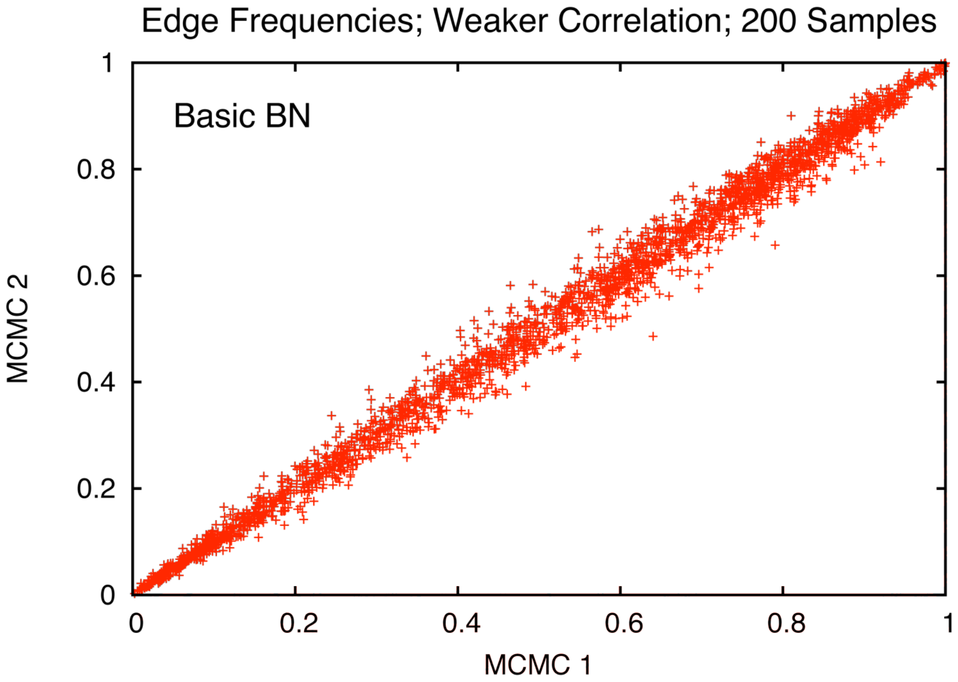

3b)

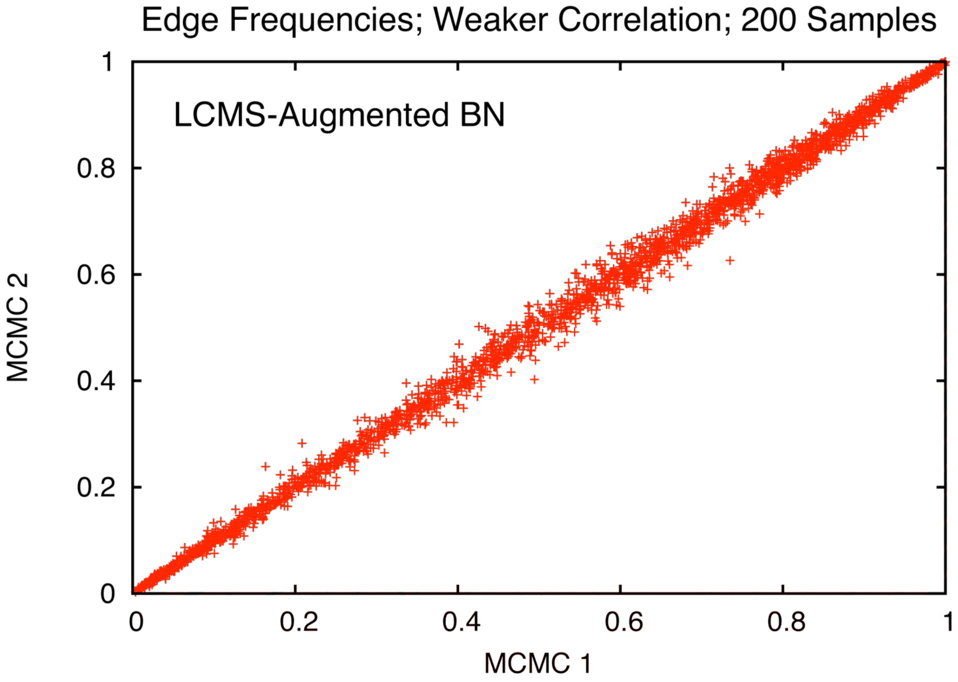

3c)

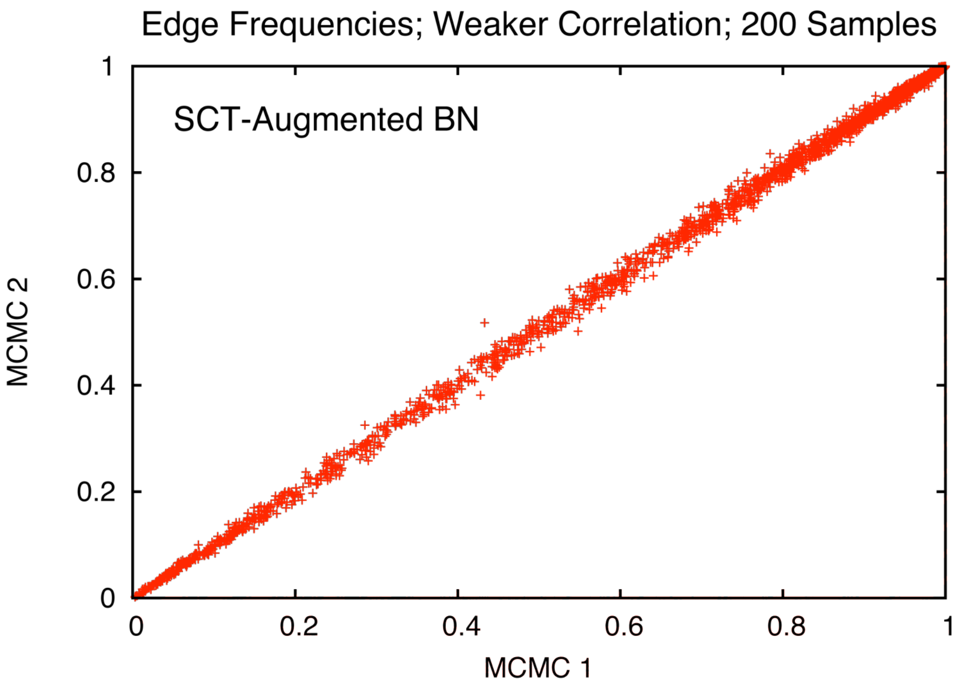

Supplementary figures 3a-3c, which correspond to supplementary figures 1 and 2, depict edge frequencies from two independent MCMC runs for each of the three methods. We plot all 2,598 edges from the true network used to simulate the data. In S3a, the unaugmented Bayesian networks have slightly poorer consistency in edge frequencies as compared to the SCT-augmented method depicted in S3c, reflecting the fact that they converge at a slightly lower rate (see Supplementary figure 2).

Supplementary Tables 1a-b.

a) Regression

		Power			
		1	2	3	4
Conditional Weight	0.0	0.600	0.652	0.643	0.635
	1.0	0.630	0.692	0.698	0.668
	2.0	0.662	0.722	0.726	0.700
	3.0	0.677	0.739	0.746	0.722
	4.0	0.698	0.772	0.762	0.738
	5.0	0.714	0.768	0.778	0.750
	6.0	0.727	0.798	0.786	0.782

b) PCC

		Power			
		1	2	3	4
Conditional Weight	0.0	0.587	0.636	0.630	0.603
	1.0	0.629	0.692	0.705	0.679
	2.0	0.668	0.732	0.744	0.744
	3.0	0.730	0.782	0.774	0.775
	4.0	0.706	0.785	0.783	0.787
	5.0	0.715	0.792	0.788	0.790
	6.0	0.721	0.800	0.792	0.791

Supplementary tables 1a and 1b, which derive from the dataset composed of weaker correlation structure and 200 samples, present AUC values for the SCT method when implemented with (a) regression functions and (b) Pearson's correlation coefficient. The results indicate that both approaches yield comparable levels of performance. Ultimately we employed regression functions since they are suitable for modeling interactions between binary (loci) and continuous (expression) variables. Furthermore, the regression functions can be adapted to studies involving diploids that require the modeling of heterozygosity.

Supplementary Tables 2a-b.

Sample Standard Deviations for the Conditional Potential

a) **Strong Correlation**

	100 S	200 S	300 S
**True Networks**	0.055	0.049	0.046
**Linear Triplets**	0.054	0.040	0.030

b) **Weak Correlation**

	100 S	200 S	300 S
**True Networks**	0.054	0.040	0.038
**Linear Triplets**	0.049	0.035	0.028

Supplementary table 2 compares the standard deviations obtained from sampling on the true networks as well as those obtained by measuring the conditional potential for 10,000 randomly generated linear triplets. For the true networks, given knowledge of the structure, we randomly select triplets and measure the conditional potential. Generally, we want to approximate these values without prior knowledge of the structure. To this end, we employed a simple sampling procedure with randomly generated data for linear triplets, represented graphically as $x \rightarrow y \rightarrow z$. Each of the 10,000 iterations involves a new triplet. For each iteration, we first generate data for **x** with random values taken from a standard normal distribution N~(0,1). Next, we generate data for **y** via the linear model $\Sigma y_i = a_i x_i + \varepsilon_i$, where a_i is selected from N~(0.75, 0.2) or N~(0.6, 0.2) for the strongly and weakly correlated data, respectively. ε_i is selected from N~(0, 1). Similarly, data for **z** is generated from **y** with the linear model $\Sigma z_i = a_i y_i + \varepsilon_i$. This simple method generally underestimates the true standard deviations, which probably reflects the fact that true networks have more complicated motifs. This suggests that more accurate estimates might be obtained if one wishes to assume the presence of more complex motifs (e.g. 4-mers with v-structures and feed-forward motifs) and adjust the sampling procedure accordingly. In our study, for a given number of samples, we averaged values between the strongly and weakly correlated datasets. For example, for datasets with 100 samples, we used a SD of (0.054 + 0.049)/2 = 0.052 for both the strongly and weakly correlated datasets.
